# The Impact of Multidisciplinary Team Meetings on the Survival of Stage IV NSCLC Patients

**DOI:** 10.1002/cam4.71350

**Published:** 2025-11-02

**Authors:** Zhengyang Lu, Nan Zhang, Qian Gao, Pengfei Li, Weimin Guan, Shihong Lin, Wenxuan Yan, Boyu Liu, Youhua Lu, Jinming Yu

**Affiliations:** ^1^ School of Public Health Shandong Second Medical University Weifang China; ^2^ Cancer Hospital of Shandong First Medical University (Shandong Cancer Institute, Shandong Cancer Hospital) Jinan China; ^3^ School of Public Health Administration Shandong First Medical University & Shandong Academy of Medical Science Jinan China

**Keywords:** multidisciplinary team, multidisciplinary team meetings, non‐small cell lung cancer, survival

## Abstract

**Background:**

With treatment strategies for cancer constantly evolving, the multidisciplinary team (MDT) plays a key role in optimizing cancer care, but its real‐world impact on survival remains unclear. This study aims to examine the impact of MDT on overall survival (OS) among patients with stage IV non‐small cell lung cancer (NSCLC), based on real‐world data including all eligible patients.

**Method:**

Patients with stage IV NSCLC who were admitted for the first time to Shandong Cancer Hospital from January 1, 2021 to December 31, 2021 were divided into MDT group and non‐MDT group according to MDT meeting involvement. Follow‐up period extended from January 1, 2021, to May 31, 2024. Kaplan–Meier curves and log‐rank tests were used to analyze survival differences, while Cox proportional hazards models were employed to identify factors associated with overall survival.

**Result:**

One thousand six hundred and sixty‐four patients were included. No statistical differences were found between the MDT group (*n* = 1238) and the Non‐MDT group (*n* = 426) in baseline characteristics, but differences were observed in treatment modalities. The MDT group exhibited a longer median overall survival compared to the Non‐MDT group (26.25 vs. 21.42 months; log‐rank *χ* ^*2*^  = 4.93, *p* = 0.03). Furthermore, MDT was associated with a reduction in the risk of mortality (adjusted HR = 0.86; 95% CI, 0.75–0.99; *p* < 0.05).

**Conclusion:**

MDT can effectively reduce mortality risk. Future implementation requires recognizing its potential additional benefits and developing tailored strategies.

## 1 Introduction

Lung cancer has become one of the leading causes of cancer‐related mortality worldwide for both men and women [1]. Among these, approximately 85% are non‐small cell lung cancers (NSCLC), which account for nearly one‐fifth of all cancer deaths globally [2]. Despite advances in early detection and standard treatment, NSCLC is often diagnosed at an advanced stage, with poor prognosis [3]. The management of NSCLC has become increasingly complex, necessitating a collaborative approach where specialists from multiple disciplines make joint decisions to optimize patient care [4]. Multidisciplinary teams (MDTs) are groups of professionals from various disciplines who collaborate regularly to make treatment decisions, enabling more effective and timely diagnosis and treatment pathways for patients [5].

Advancements in lung cancer treatment have continuously evolved in recent years, with targeted therapy and immunotherapy now complementing traditional methods such as chemotherapy, radiotherapy, and surgery as essential components of disease management, leading to substantial improvements in patient prognosis. This progress has also expanded the therapeutic landscape for non‐small cell lung cancer (NSCLC), where breakthroughs in treatment have markedly enhanced both diagnosis and care for NSCLC patients [6, 7, 8, 9, 10].

Numerous studies have demonstrated the positive impact of MDT on improving overall survival (OS) for patients with lung cancer [11, 12, 13]. As the diagnosis and treatment of lung cancer become increasingly complex, leveraging the broad expertise within MDT to design the most effective treatment plans can yield the greatest clinical benefits [14]. Consequently, MDT is now regarded as a cornerstone of lung cancer management [15].

However, the effectiveness of MDT in the context of emerging treatment paradigms still requires further exploration and clarification. Most existing studies on MDT effectiveness focus on selected subgroups or matched cohorts, but due to variations in MDT inclusion criteria—such as differences in patient characteristics and institutional inclusion standards—it remains challenging to conduct randomized controlled trials (RCTs) for proving the effectiveness of MDT [16, 17, 18]. To address these gaps, real‐world evidence may offer valuable insights into its potential impact on advanced cancer care.

Shandong Cancer Hospital has progressively implemented MDT meetings since March 2020. In 2021, the hospital began encouraging the inclusion of all stage IV NSCLC cases—except those with clearly curative options such as surgery—into MDT discussions held once or twice weekly before treatment initiation. These MDT services are provided free of charge to ensure accessibility. During each meeting, the responsible physician presents the patient's clinical information and proposed treatment plan, which is then reviewed and optimized by the multidisciplinary team. Hospital administration sets minimum submission quotas and randomly selects a portion of inpatients for mandatory MDT discussion.

Therefore, this study aims to provide a comprehensive real‐world assessment of the impact of MDT on survival outcomes in patients with stage IV non‐small cell lung cancer, by comparing all patients who were discussed in MDT meetings with those who were not.

## 2 Material and Method

### 2.1 Study Design and Patients

This study was conducted at Shandong Cancer Hospital, where all cases of stage IV NSCLC patients are encouraged to be discussed in MDT meetings. We retrospectively identified all newly admitted patients diagnosed with stage IV NSCLC in 2021, with admission dates ranging from January 1 to December 31. Patients whose cases were discussed in MDT meetings were classified as the MDT group, while those who were not were assigned to the Non‐MDT group, based on hospital administrative records. Cases not discussed in MDT meetings (Non‐MDT group) include those where MDT implementation was insufficient (e.g., a lack of diverse experts, an inadequate number of participants, or delays in meetings), patients who received treatment solely within individual departments, and patients who left the hospital after receiving symptomatic treatment or diagnostic testing. The overall survival between the two groups based on this classification was then compared.

### 2.2 Data Collection

Information on MDT discussion (whether the case was presented in lung cancer MDT meetings) was obtained from records maintained by the Medical Affairs Department, which oversees the implementation of MDT. Patient demographics (age, gender, insurance types), lifestyle factors (smoking and alcohol consumption), clinical information (presence of chronic diseases, family history, treatment‐naive, and pathological type), as well as treatment data (whether to adopt chemotherapy, radiotherapy, targeted therapy, immunotherapy, or surgery) were collected from medical records retrieved from the hospital information system.

Survival data were obtained through a combination of passive and active follow‐up methods. A passive follow‐up was conducted by retrieving the identification information of patients newly admitted in 2021 from the hospital information system. Using patient IDs, we then matched these cases with mortality data from the Shandong Center for Disease Control and Prevention (CDC), the official provincial‐level public health authority, to obtain survival status up to May 31, 2024. During active follow‐up, attention was directed toward cases from the Shandong CDC data that lacked recorded mortality outcomes. Telephone follow‐ups were conducted to determine whether patients had experienced mortality events and, if so, to ascertain the time of those events. In cases where families were unable to confirm the occurrence of death, these instances were classified as censored in this study. For patients with a confirmed death but missing time of death, multiple imputation was performed to estimate survival times based on clinical characteristics, including age, sex, pathological diagnosis, chronic diseases, and death status.

The follow‐up period in this study was defined based on the newly admitted dates in 2021, with the follow‐up end date set at May 31, 2024. Events were defined as instances of patient death occurring within the observation period, with the date of death marking the end of observation. For patients without a recorded death, the end of observation was set at the follow‐up end date of May 31, 2024, and these cases were classified as censored. The survival observation period spanned from January 1, 2021, to May 31, 2024, totaling 1246 days.

### 2.3 Statistical Analysis

Chi‐square tests and the Wilcoxon signed‐rank test were used to compare differences between the MDT and Non‐MDT groups. Missing survival times were addressed using multiple imputation. Survival curves were generated with the Kaplan–Meier method, and survival differences were assessed using the log‐rank test. A Cox proportional hazards (CPH) model was employed to evaluate factors affecting survival rates, adjusting for the impact of MDT meetings on mortality risk. Hazard ratios (HRs) and 95% confidence intervals (CIs) were reported, with a significance level of α = 0.05. All statistical analyses were conducted using R version 4.3.0.

## 3 Results

### 3.1 Patient and Disease Characteristics

One thousand six hundred and sixty‐four patients were included, with 426 in the Non‐MDT group and 1238 in the MDT group. Patients' baseline characteristics are presented in Table 1. The median age of Non‐MDT patients was 63 years (range: 55–70), while the MDT group had a median age of 63 years (range: 55–69). There were a total of 975 males and 689 females. Regarding insurance types, 1054 patients had urban resident insurance, 531 had urban employee insurance, and 79 were classified as other. Among the patients, 613 had chronic diseases, while 1051 had no other underlying diseases.

**TABLE 1:** Descriptive characteristics of the patients.

Characteristics	MDT		*χ2*/*z*	*p*
	MDT (*n* = 1238)	Non‐MDT (*n* = 426)		
Sex			1.75	0.19
Male	737 (59.53)	238 (55.87)		
Female	501 (40.47)	188 (44.13)		
Age	63 (55–69)	63 (55–70)	0.80	0.42
Insurance			1.70	0.43
UR	790 (63.81)	264 (61.97)		
UE	394 (31.83)	137 (32.16)		
Others	54 (4.36)	25 (5.87)		
Smoke			1.43	0.23
Yes	485 (39.18)	153 (35.92)		
No	753 (60.82)	273 (64.08)		
Alcohol			0.24	0.62
Yes	326 (26.33)	107 (25.12)		
No	912 (73.67)	319 (74.88)		
Family history			0.06	0.80
Yes	114 (9.21)	41 (9.62)		
No	1124 (90.79)	385 (90.38)		
Treatment‐naive			0.04	0.83
Yes	270 (21.81)	95 (22.30)		
No	968 (78.19)	331 (77.70)		
Chronic diseases			0.12	0.73
Yes	459 (37.08)	154 (36.15)		
No	779 (62.92)	272 (63.85)		
Pathology			2.20	0.33
SCC	154 (12.44)	63 (14.79)		
Adenocarcinoma	1049 (84.73)	348 (81.69)		
Others	35 (2.83)	15 (3.52)		

*Note:* (*) MDT, the patient group whose cases were discussed at the MDT meetings; Non‐MDT, the patient group whose cases have not been discussed in the MDT meetings, or in a form that cannot meet the MDT meeting's standards. UR, Urban resident medical insurance, which includes New Rural Cooperative Medical Insurance; UE, Urban employee medical insurance; Others include commercial insurance and out‐of‐pocket expenses. SCC is short for Squamous cell carcinoma; Treatment‐naive refers to not receiving any anti‐tumor treatment before being admitted to the hospital.

There were no statistically significant differences in terms of age, sex, insurance type, smoking, alcohol consumption, family history, treatment‐naive, chronic diseases, and pathological characteristics (age was tested using the Wilcoxon test, and other characteristics with the chi‐square test).

However, statistically significant differences were observed in treatment modalities determined after MDT discussion (Table 2), including the use of chemotherapy, radiotherapy, targeted therapy, and surgery. Compared to the non‐MDT group, the MDT group had a higher proportion of patients receiving chemotherapy, targeted therapy, and radiotherapy, while fewer patients underwent surgery. Additionally, a greater proportion of patients in the MDT group received treatment during their first admission compared to the non‐MDT group.

**TABLE 2:** Treatment modalities determined after MDT discussion.

Characteristics	MDT		*χ2*/*z*	*p*
	MDT (*n* = 1238)	Non‐MDT(*n* = 426)		
Chemotherapy			13.87	< 0.01
Yes	730 (58.97)	207 (48.59)		
No	508 (41.03)	219 (51.41)		
Targeted			19.63	< 0.01
Yes	651 (52.58)	171 (40.14)		
No	587 (47.42)	255 (59.86)		
Radiotherapy			34.83	< 0.01
Yes	390 (31.50)	71 (16.67)		
No	848 (68.50)	355 (83.33)		
Surgery			46.02	< 0.01
Yes	21 (1.70)	37 (8.69)		
No	1217 (98.30)	389 (91.31)		
Immunotherapy			1.01	0.31
Yes	141 (11.39)	41 (9.62)		
No	1097 (88.61)	385 (90.38)		

### 3.2 Survival

**FIGURE 1:**
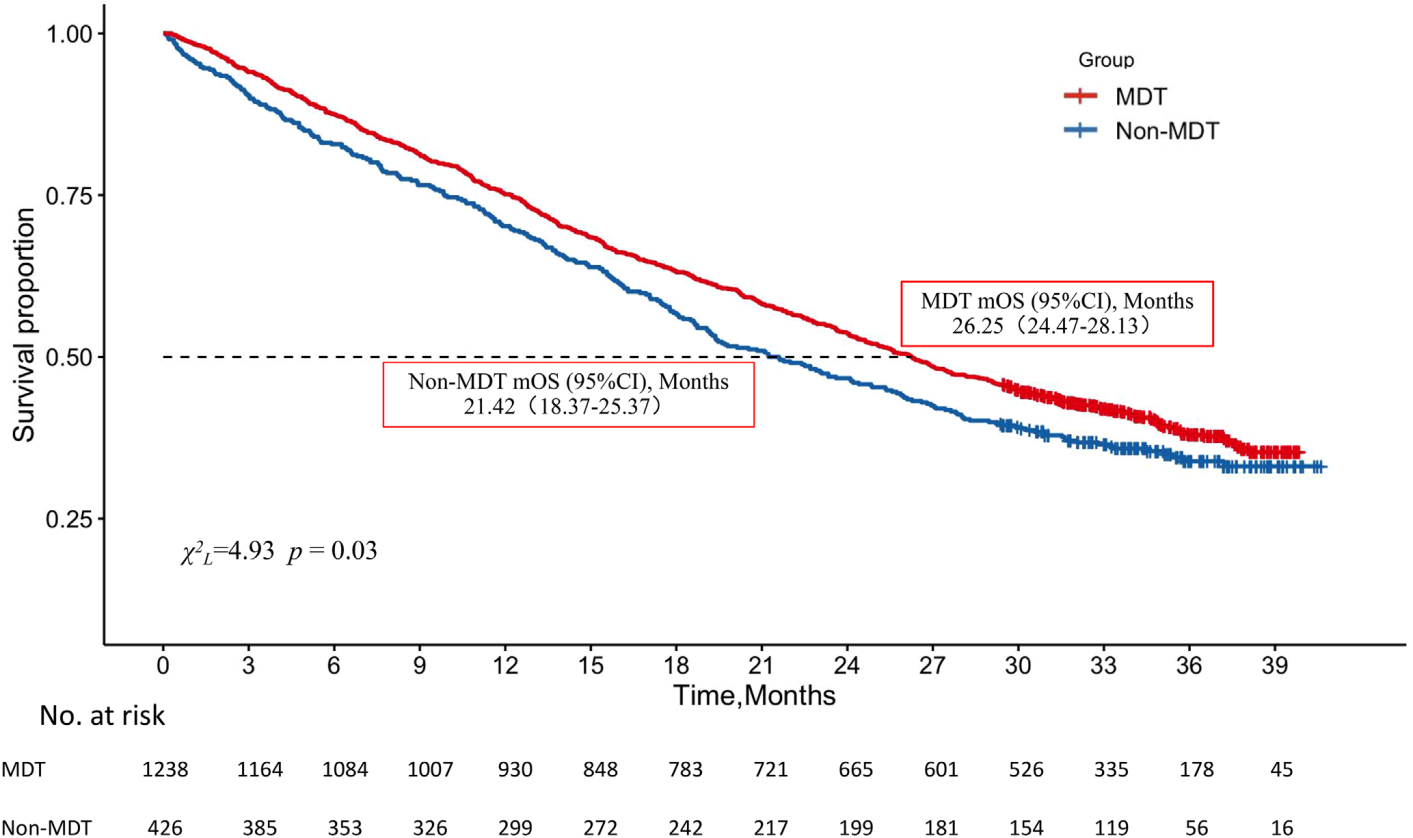
Overall Survival. Shown is the Kaplan–Meier estimate of overall survival of patients with IV NSCLC. Censored data are indicated by tick marks. Patients who do not have a death record in the death data of the Centers for Disease Control and Prevention, and those who are confirmed to have not died or cannot be contacted through follow‐up, are considered as censored data. A statistically significant difference in overall survival was observed between the MDT and Non‐MDT groups (*χ* ^*2*^ *L* = 4.93, *p* = 0.03), with the MDT group showing a lower risk of mortality (HR = 0.86; 95% CI, 0.74 to 0.98).

By the end of the follow‐up period, out of 1664 patients, there were a total of 1019 (61.24%) death events reported: 743 (60.02%) in the MDT group and 276 (64.79%) in the Non‐MDT group (Figure 1). The median survival for patients in the MDT group was 26.25 months (95% CI, 24.47 to 28.13), compared to 21.42 months (95% CI, 18.37 to 25.37) for the Non‐MDT group. Overall, the survival outcomes were statistically significantly better in the MDT group than in the Non‐MDT group (*χ* ^*2*^ *L* = 4.93, *p* = 0.03), with a hazard ratio of 0.86 (95% CI, 0.74 to 0.98).

MDT group demonstrated higher survival rates than the Non‐MDT group across different genders and in patients with or without chronic diseases (at 1, 2, and 3 years postdiagnosis) (Table 3). In age groups under 60 years and between 61 and 69 years, the MDT group also exhibited higher survival rates (at 1, 2, and 3 years postdiagnosis). However, in patients over 71 years of age, the 1 and 2‐year survival rates were higher in the MDT group, while the 3‐year survival rate was lower compared to the Non‐MDT group.

**TABLE 3:** OS rates for different groups.

Characteristic	Year after diagnosis	MDT (95% CI)	Non‐MDT (95% CI)
Age group			
≤ 60	1	0.82 (0.79–0.86)	0.75 (0.69–0.81)
	2	0.62 (0.58–0.66)	0.55 (0.48–0.63)
	3	0.47 (0.42–0.52)	0.41 (0.34–0.49)
61–70	1	0.73 (0.70–0.78)	0.69 (0.62–0.77)
	2	0.51 (0.47–0.56)	0.43 (0.35–0.51)
	3	0.38 (0.33–0.43)	0.33 (0.27–0.42)
≥ 71	1	0.61 (0.55–0.68)	0.62 (0.53–0.73)
	2	0.35 (0.29–0.42)	0.34 (0.25–0.45)
	3	0.18 (0.13–0.25)	0.22 (0.15–0.33)
Sex			
Female	1	0.83 (0.80–0.86)	0.81 (0.75–0.87)
	2	0.59 (0.55–0.63)	0.56 (0.49–0.63)
	3	0.45 (0.41–0.50)	0.37 (0.31–0.45)
Male	1	0.70 (0.67–0.73)	0.61 (0.55–0.68)
	2	0.49 (0.45–0.53)	0.38 (0.33–0.45)
	3	0.33 (0.30–0.37)	0.31 (0.26–0.38)
Chronic diseases			
No	1	0.76 (0.73–0.79)	0.73 (0.68–0.78)
	2	0.54 (0.50–0.57)	0.49 (0.43–0.55)
	3	0.39 (0.36–0.43)	0.36 (0.31–0.43)
Yes	1	0.73 (0.69–0.77)	0.65 (0.58–0.73)
	2	0.52 (0.47–0.56)	0.41 (0.34–0.49)
	3	0.35 (0.31–0.41)	0.29 (0.23–0.38)

### 3.3 Cox Regression

**FIGURE 2:**
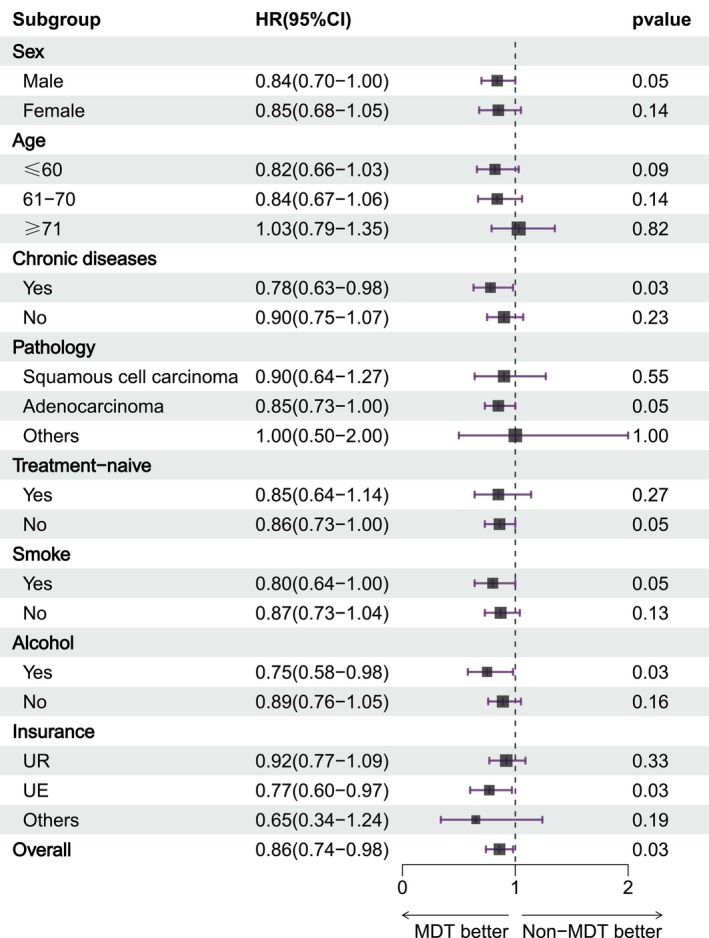
Forest Plot of Univariate Cox Regression. Shown is a forest plot of the subgroup analysis conducted using the Cox proportional hazards model. A hazard ratio (HR) less than 1.00 indicates a reduced risk of mortality for patients in the MDT group compared to those in the Non‐MDT group. Except for the age group over 71 years (HR = 1.03; 95% CI, 0.79–1.35) and patients with other pathological types (HR = 1.00; 95% CI, 0.50–2.00), the MDT group showed a trend toward lower mortality risk across most subgroups.

Forest plot (Figure 2) indicated that the overall survival benefits of the MDT group compared to the Non‐MDT group were consistent across most predefined subgroups, although the degree of benefit varied. The confidence intervals overlapped both within and between subgroups. Notably, except for the age group over 71 years (HR = 1.03; 95% CI, 0.79–1.35) and other pathological types (HR = 1.00; 95% CI, 0.50–2.00), the MDT group consistently exhibited a lower risk of mortality compared to the Non‐MDT group across other subgroups.

Univariate Cox regression analysis indicated that involvement in MDT meetings was associated with lower risk of mortality (HR = 0.86; 95% CI, 0.75–0.98). In contrast, higher age, male gender, smoking, alcohol consumption, presence of chronic diseases, and adenocarcinoma were all linked to an increased mortality risk (*p* < 0.05). Multivariate Cox regression analysis (Table 4) identified MDT involvement, age, male (versus female), and adenocarcinoma (versus squamous cell carcinoma) as independent factors influencing mortality risk. Specifically, patients involved in MDT meetings demonstrated a statistically significant lower risk of mortality compared to the Non‐MDT group (HR = 0.86; 95% CI, 0.75–0.99; *p* < 0.05). Male patients had a 1.3‐fold increased risk of death compared to female patients (HR = 1.30; 95% CI, 1.11–1.53; *p* < 0.01). Additionally, for each one‐year increase in age, the risk of mortality increased by 1.03 times (HR = 1.03; 95% CI, 1.02–1.04; *p* < 0.01). Furthermore, patients with adenocarcinoma exhibited a lower risk of death compared to those with squamous cell carcinoma (HR = 0.73; 95% CI, 0.61–0.88; *p* < 0.01).

**TABLE 4:** Multivariate Cox regression.

Characteristics	Reference class class	coef	univariate HR (95% CI)	*p*	coef	multivariate HR (95% CI)	*p*
MDT involvement							
Yes	No	−0.16	0.86 (0.75–0.98)	< 0.05	−0.15	0.86 (0.75–0.99)	< 0.05
Age		0.03	1.03 (1.03–1.04)	< 0.01	0.03	1.03 (1.02–1.04)	< 0.01
Sex							
Male	Female	0.35	1.41 (1.25–1.61)	< 0.01	0.26	1.30 (1.11–1.53)	< 0.01
Smoke							
Yes	No	0.28	1.32 (1.17–1.50)	< 0.01	0.05	1.05 (0.88–1.26)	0.57
Alcohol							
Yes	No	0.16	1.18 (1.03–1.35)	< 0.05	−0.08	0.92 (0.77–1.10)	0.38
Chronic diseases							
Yes	No	0.13	1.14 (1.00–1.29)	< 0.05	0.07	1.07 (0.94–1.22)	0.30
Pathology							
Adenocarcinoma	SCC	−0.54	0.58 (0.49–0.69)	< 0.01	−0.31	0.73 (0.61–0.88)	< 0.01
Others		−0.03	0.97 (0.68–1.38)	0.86	0.09	1.09 (0.76–1.56)	0.63
Insurance							
UR	UE	−0.05	0.95 (0.84–1.09)	0.49			
Others		−0.30	0.74 (0.54–1.02)	0.07			
Treatment‐naive							
Yes	No	0.14	1.15 (1.00–1.33)	0.06			

*Note:* (*) SCC: Squamous cell carcinoma.

## 4 Discussion

This study utilized both active and passive follow‐up methods to comprehensively assess the impact of MDT involvement on the overall survival (OS) of stage IV NSCLC patients treated at Shandong Cancer Hospital in 2021. By including all eligible patients within a full year, rather than focusing on selected subgroups or matched samples, the study reflects a real‐world, comprehensive approach to evaluating MDT effectiveness. Baseline characteristics, MDT inclusion, and treatment information were collected to support a robust comparison. No statistically significant differences were observed in demographics, lifestyle factors, or clinical characteristics between the MDT and non‐MDT groups, indicating comparability between the two groups.

In multivariate analysis, MDT involvement emerged as an independent factor for improved survival, associated with a 14% reduction in mortality risk (HR = 0.86; 95% CI, 0.75–0.99), consistent with prior findings on the prognostic benefits of MDT involvement [19, 20, 21, 22]. Although the survival benefit of MDT was generally observed across subgroups (Table 3, Figure 2), the group aged over 71 years showed a non‐significant trend toward higher mortality in the MDT group, potentially due to age‐related limitations in treatment tolerance [23].

Patient survival times in this study were relatively longer than those reported in previous research. Justin Yeh's 2018 study reported a median survival of 12 months for metastatic NSCLC and an overall 5‐year lung cancer survival rate of approximately 20% [24, 25], which contrasts with the longer median survival observed in our study (MDT: 26.25 months; Non‐MDT: 21.42 months). This difference may reflect recent advances in diagnostic techniques and treatment modalities for NSCLC, which have contributed to improved patient outcomes, as supported by multiple studies [9, 10, 24, 26]. Slight delays in death reporting may have occurred in a minority of cases due to administrative processing, delayed certification, or late notification by family members, even though mortality data were obtained from official records provided by the Shandong CDC.

The change in treatment approach may be one of the pathways through which MDT meetings influence patient survival. Statistically significant differences were observed between the MDT and non‐MDT groups in terms of treatment strategies (Table 2), with the MDT group overall demonstrating better survival outcomes. A previous study has also noted that patients discussed in MDT meetings are more likely to receive accurate and complete preoperative staging as well as neoadjuvant or adjuvant therapies [16]. MDT involvement is also associated with an increased likelihood of confirming histological diagnoses, accurate clinical staging, and receiving surgical treatment [11]. This underscores the role of MDT in improving survival by facilitating precise diagnosis and the adoption of scientifically grounded treatment strategies. Notably, for patients with stage IV NSCLC, surgical interventions were primarily palliative, aimed at symptom management and addressing complications related to distant metastases, rather than achieving curative outcomes. Moreover, some hospitalized patients may receive symptomatic treatment or undergo pathological examinations without continuing anti‐tumor therapy within the hospital, possibly due to patients' or families' decisions to seek care at other institutions or financial constraints that may lead to treatment discontinuation [27, 28, 29].

Recent studies have shown that the benefits of the MDT extend beyond survival outcomes, suggesting that its impact on survival may not solely be attributed to changes in treatment approaches. MDT optimizes the patient care process by reducing waiting times for consultations, surgery, and hospitalization, while also increasing the continuity and coordination of healthcare services, thereby improving the overall healthcare delivery time frame [17, 30, 31]. These findings highlight the systemic and complex nature of MDT's effectiveness. Incorporating a broader range of outcome variables into a systematic evaluation of MDT could be key to capturing its full impact.

Although MDTs have become standard practice in many centers, high‐quality evidence supporting their survival benefits remains limited, partly due to the challenges of conducting randomized controlled trials in this context [2, 16, 17, 18]. Several factors contribute to the difficulty in evaluating the effectiveness of MDTs. One major challenge is the inconsistency between MDT recommendations and their actual implementation in clinical practice, often influenced by individual patient preferences or family decisions [32, 33]. Furthermore, there is substantial heterogeneity in how MDTs are structured and operated across institutions—including variations in format (in‐person vs. network‐based), expert composition, case inclusion criteria, and administrative support—which can lead to variable outcomes [15, 34, 35]. In addition, data quality and consistency pose significant limitations; discrepancies in clinical databases—such as inaccurate or missing diagnostic and death dates—have been shown to substantially impact survival estimates, thus hindering the validity of multi‐center analyses [36, 37].

This study has several limitations. As a single‐center analysis based on data from Shandong Cancer Hospital, the findings primarily reflect the characteristics of MDT implementation at this institution, which may limit their generalizability. Follow‐up was uniformly conducted on May 31, 2024, resulting in censored data clustering beyond the median survival time, which may affect survival estimates. Due to technical constraints, we could not assess the use of combined treatment modalities or the implementation of MDT recommendations. Although individual treatment data were collected, these do not fully reflect the complexity of treatment strategies. While studies indicate that most patients follow MDT recommendations, the translation of these recommendations into clinical practice can vary due to individual patient and family decisions, which may in turn influence overall survival outcomes [32].

## 5 Conclusion

MDT is independently associated with a reduced mortality risk in stage IV NSCLC patients. The wider application and promotion of MDT require incorporating its broader benefits into the evaluation system, and conducting research on the influencing factors of MDT implementation to identify the most suitable mode for specific medical institutions.

## Author Contributions

The conceptual and primary design of the study was performed by J.Y. and Y.L. Material preparation, data collection, and analysis were performed by Z.L., N.Z., Q.G., and the results were interpreted. Z.L. drafted the manuscript, and N.Z., Q.G., P.L., W.G., S.L., W.Y., and B.L. reviewed it critically for important intellectual content. J.Y. and Y.L. gave final approval of the version to be published.

## Ethics Statement

The study was approved by the Institutional Ethical Review Board of Shandong Cancer Hospital and Institute (Number: SDTHEC2024001054). The IRB granted a waiver of informed consent in accordance with institutional and ethical guidelines.

## Conflicts of Interest

The authors declare no conflicts of interest.

## Data Availability

The data that support the findings of this study are available from the corresponding author upon reasonable request.
